# Composing egocentric and allocentric maps for flexible navigation

**DOI:** 10.1371/journal.pcbi.1013905

**Published:** 2026-01-23

**Authors:** Daniel Shani, Peter Dayan

**Affiliations:** 1 Max Planck Institute for Biological Cybernetics, Tübingen, Germany; 2 University of Tübingen, Tübingen, Germany; 3 Sainsbury Wellcome Centre, UCL, London, United Kingdom; UT Austin: The University of Texas at Austin, UNITED STATES OF AMERICA

## Abstract

Egocentric representations of the environment have historically been relegated to being used only for simple forms of spatial behaviour such as stimulus-response learning. However, in the many cases that critical aspects of policies are best defined relative to the self, egocentric representations can be advantageous. Furthermore, there is evidence that forms of egocentric representation might exist in the wider hippocampal formation. Nevertheless, egocentric representations have yet to be fully incorporated as a component of modern navigational methods. Here we investigate egocentric successor representations (SRs) and their combination with allocentric representations. We build a reinforcement learning agent that combines an egocentric SR with a conventional allocentric SR to navigate complex 2D environments. We demonstrate that the agent learns generalisable egocentric and allocentric value functions which, even when only additively composed, allow it to learn policies efficiently and to adapt to new environments quickly. Our work shows the benefit for egocentric relational structure to be captured, as well as allocentric. We offer a new perspective on how cognitive maps could usefully be composed from multiple simple maps representing associations between state features defined in different reference frames.

## Author summary

Humans and animals use two kinds of representation to navigate. One is world-centered or allocentric: a bird’s-eye view of objects and places within which the subject is positioned. The other is self-centered or egocentric: with the locations of objects and places being referenced to the subject themselves, e.g., “the wall on my right.” Most neuroscientific research into navigation leans heavily on allocentric representations, likening them to “maps” that represent associations between objects and places and can facilitate complex planning. Meanwhile egocentric representations are viewed as being rather simple, and mainly employed for stimulus-response learning. Here, we show the benefits of constructing and using a second, egocentric, map alongside the conventional, allocentric, one. We designed a simple learning agent that builds both sorts of map, and uses them cooperatively. The egocentric map captures local rules that repeat across different layouts of environments, while the allocentric map handles overall direction. Since the local rules are reusable, the combined agent adapts faster than a purely allocentric one when environments change: it needs fewer trials to find good paths and avoids getting “trapped” by obstacles. Our approach highlights the benefit for planning of using multiple maps in different reference frames.

## 1 Introduction

One of the most important dichotomies in spatial understanding is that between allocentric versus egocentric representations. Allocentric representations are tied to a reference frame in the outside world, as if there was a form of (at least contextually local) compass. By contrast, egocentric representations are tied to one of a collection of personal reference frames. In work linking spatial representations to spatial behavior [1,2], the underlying difference has historically been very closely tied to that between map-based strategies [3] versus taxon-like habits, built out of motor routines and sensory data [4,5].

There has been a huge wealth of work on allocentricity in spatial processing. Behavioural evidence for the existence of such representations is broad and deep, including a host of experiments that demonstrate the ability of animals to infer shortcuts [3,6]. These experiments, as well as others that show that rodents use geometric features of their environment to reorient themselves [7], and evidence of path integration [8,9], led to the suggestion that a geometric module exists in the brain [10,11]. Such a module would process the external environment, turning sensory information which is inevitably egocentric into an allocentric form that is then useful, for instance, for navigation (whence allocentric choices have to be converted back into egocentric coordinates to determine movement). However, the notion that this geometric module might play a role in other spatial tasks, such as representing object locations, was not prevalent. Later work, demonstrating the use of external cues for object memory, suggested the possibility of allocentric spatial memory [12]. Abstracting away from the domain of space, there has also been work on allocentric views of social hierarchies [13]. Equally, most work on predictive representations such as the successor representation (SR; [14]) or the default representation [15] has been conducted in allocentric terms.

Allocentric processing is also important for artificial systems. For instance, there is an abundance of work on simultaneous localization and mapping (SLAM; [16]), which concerns the problem of building an allocentric representation of the environment while navigating in it. It is again necessary to ground the map in egocentric inputs to infer odometry measurements. Early SLAM paradigms [17] made use of the Extended Kalman Filter [18,19] to represent uncertain relationships between different features of the environment. Current neuroscientific models of cognitive maps [20–23] have retained this classical SLAM-like perspective that embeds objects in an allocentric cognitive map, at most using egocentric representations to build such a map which is then used for planning.

Egocentricity has somewhat more sporadically been seen as important for navigational processing. Human and animal studies [10,24] highlight various phenomena implying the use of an egocentric reference frame. A prominent piece of evidence for such an egocentric representation is the presence of alignment effects – in studies where participants had to learn the location of objects from a single perspective and then recognise those configurations from novel perspectives, the recognition time increased linearly in the angular distance between the two viewpoints [25]. Similarly when participants had to point to the imagined relative position of an object from an imagined viewpoint, they responded faster and more precisely when the imagined viewpoint was aligned with the learned viewpoint [26]. However, current neuroscientific models [1] have equated these egocentric representations with stimulus-response learning, as in a taxon strategy, rather than using them for the sort of planning that is associated with allocentric representations.

Nevertheless, egocentric representations can also be used in planning. One early influential line of work along these lines suggested the notion of so-called deictic representations [27]. These are locally indexed to the agent – a classic example being ’the object *in my left hand*’ – and have the advantages and disadvantages of automatic generalization (to any other object in the same hand). Deictic representations have attracted some attention in the field of reinforcement learning [27,28]. For instance, because of the inherent generalization they afford, they were considered as potential contributors to model-based planning in a partially observable Markov decision process (POMDP). Unfortunately, the results of doing this were rather mixed [28] – deictic representations actually worsened learning performance when compared to a fully-propositional representation. The authors attributed this worsening to the history dependence of an optimal deictic policy – the behaviour of the agent is dependent on the task-location of the agent which is only knowable by examining its action history, and this became hard when the history included exploratory actions.

Although not originally couched in exactly deictic terms, a prominent contribution to work on hierarchical reinforcement learning, the Hierarchical Abstract Machine (HAM) from [29], can be interpreted as such. HAMs are typically modest-sized state machines that specify separable and composable parts of a policy. [29] considered environments in which there are many, identical, obstacles around which an agent has to navigate on the way to a distant goal. In this case, a single HAM for avoiding an obstacle can be repurposed to avoid the same obstacle elsewhere. If the HAM is specified deictically (‘turn left when the wall on my right disappears’), then it will typically generalize immediately to other instances of the same object even when rotated.

We so far lack a computationally thorough investigation into modern navigational concepts that exploit egocentric representations in environments that might benefit from them. Given the power of allocentrically-based successor representations (SRs), we investigate egocentric SRs and their use in combination with allocentric representations. We build a reinforcement learning agent that combines an egocentric successor representation with a conventional allocentric SR to navigate complex 2D environments. We demonstrate that the agent learns generalisable egocentric and allocentric value functions which, even when only additively composed, help an agent learn policies efficiently and adapt to new environments quickly. Our work shows the benefit for the brain to capture egocentric, as well as allocentric, relational structure.

## 2 Methods

### 2.1 Paradigm

**Fig 1:**
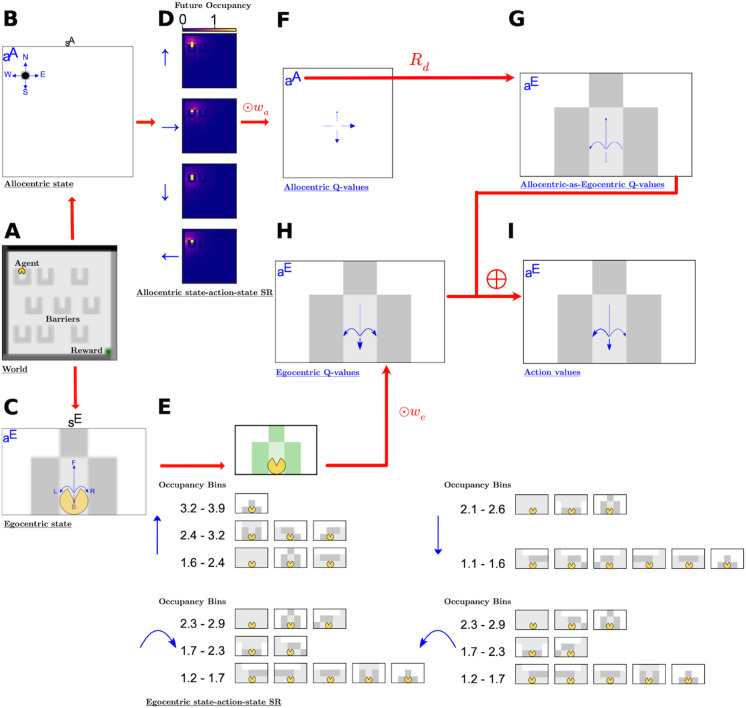
Schematic. **(A)** An example gridworld. The reward is the green box, which is in the bottom right of this gridworld. The grey patches are obstacles to movement and views. In each episode the agent starts at a random location and navigates until it reaches the reward location. The agent is denoted by a yellow pacman figure, with the possible egocentric actions shown as little blue arrows. **(B-C)** The agent receives two state representations, corresponding to its allocentric coordinates in the maze **(B)** and its egocentric view, restricted to a local window around the agent oriented in its direction **(C)** and impeded by the barriers. The repeating barriers are in light grey and then there are separate darker shades of grey for each of the four walls. The blue arrows denote the allocentric (**B**) and egocentric (**C**) action sets. **(D-E)** The agent uses the state representations to form state-action successor representations which are used as a basis of its linear function approximation of its action values. **D** shows the allocentric state-action successor representation of the state in **B**. For each allocentric action denoted in blue, the expected future state occupancies are shown as pixel intensities on the 2D grid. **E** shows the corresponding egocentric state-action SR. For each blue egocentric action, the corresponding SR is shown with descending rows denoting decreasing occupancies (in bins, with values shown as numbers on the left). Only the states with the top 60% of occupancies are shown. **(F,G,H,I)** Progressive calculation and combination of action values, where the magnitude of action values are indicated by arrow-head size. **F** shows the allocentric action-values, calculated for allocentric actions, by linear function approximation. Once these are calculated, the allocentric actions are translated, by a direction dependent rotation, into action values for the corresponding egocentric action. These allocentric-state-egocentric-action values are show in **G** and then are combined additively with the egocentric state action-values which are also calculated via linear function approximation **H**, to form our full action-values **I**.

The agent has to solve a complex maze task in which the locations and nature of the barriers and the reward change periodically. Each task involves navigating a 20×20 grid with coloured walls which contains a number of identical opaque barriers, randomly located and oriented. (Fig 1A). In each episode, the agent starts from a random location at a random orientation and moves until it finds the reward, using the actions **“go-forward”, “turn-90°-clockwise”, “turn-90°-anticlockwise”** and **“turn-180°”**. After every 1000 episodes, the walls and barriers re-organise and the reward location changes.

At each time-step the agent is supplied with allocentric and egocentric information. The allocentric information (Fig 1B) is the *s*^*A*^ = (*x*, *y*) position of the agent in the gridworld. The egocentric information (Fig 1C), *s*^*E*^, is its immediate visible view, derived from a local window of observations that is centered at the agent and oriented according to the direction the agent is facing. The egocentric view is generically aliased, by which we mean that multiple world states correspond to the same egocentric state, particularly because the barriers are opaque. To construct the egocentric state, we start with a non-obscured egocentric representation centered at the agent, that consists of all pixels in the rectangle that stretches *H* pixels in front of the agent, as well as *H* pixels to the left and right of the agent. *H* is the horizon. Then we set any pixel values which would be obscured by a block between the pixel and the agent to −∞.

### 2.2 Model

We consider a reinforcement learning agent that enjoys a geometric module for converting between egocentric and allocentric coordinate systems, and normally uses state-action SRs following both schemes. At each time-step, the agent receives both allocentric and egocentric state information and uses it to calculate the four separate SRs in each reference frame (Fig 1D and 1E). These SRs are initialised using trajectories drawn from the uniform policy – the allocentric SR from an empty gridworld while the egocentric SR from the average egocentric transition matrix across all worlds in the task. Therefore both egocentric and allocentric representations start with some prior knowledege about the mean transition structure. These SRs are then updated using temporal difference learning as the agent itself learns to behave in the environment. The state-action SRs are used as regressors for a linear function approximation of the state-action value *Q*-function. Here, the allocentric SR is associated with allocentric actions, which are automatically transformed into corresponding egocentric actions using the current head-direction of the agent. The weights of the *Q*-function are learned using *Q*-learning with ADAM [30].

### 2.3 Action selection

We denote the set of egocentric actions as

$${𝒜}^{E}=\{“go\text{-}forward”,{“turn\text{-}90}^{\circ }\text{-}clockwise”,{“turn\text{-}180}^{\circ }degree”,{“turn\text{-}90}^{\circ }\text{-}anticlockwise”\},$$ (1)

and the set of allocentric actions as

$${𝒜}^{A}=\{“North”,“East”,“South”,“West”\}.$$ (2)

At each time-step, the agent selects an egocentric action $a^{E}\in {𝒜}^{E}$ by combining information from its egocentric and allocentric SRs. For a given head-direction *d* we can define a bijection

$$R_{d}:{𝒜}^{A}\to {𝒜}^{E}$$ (3)

that maps allocentric actions to the egocentric action that would take the agent in that direction. The corresponding egocentric action is $a^{E}=R_{d}(a^{A})$.

A consequence of this is that when the allocentric component selects an allocentric action while the agent is facing a different direction, two successive choices will have to be made. Without loss of generality let the allocentric action be **“North”** while the agent faces East. In order to have the agent move North, this action will be transformed into egocentric action **“turn-90**° **-anticlockwise”** to orient the agent in the North direction, and then the allocentric component would learn that the agent has not moved allocentrically, and would furthermore have to select **“North”** again, which would this time correspond to the egocentric action **“go-forward”**, and now would move the agent to the new allocentric location. To make our model comparisons fair, we only count steps when the agent selects a **“go-forward”** action.

The agent selects a random action uniformly with probability *ϵ* and with probability 1−ϵ selects an action using a softmax policy of the action-values, with softmax temperature parameter *τ*.

### 2.4 Representations

The state-action successor representations are defined in the conventional manner as

$$M^{i}[s^{\prime }|s,a^{i}]={𝔼}_{\pi }\left[\sum_{t=0}^{\infty }\gamma_{i}^{t}I[s_{t}^{I}=s^{\prime }|s_{0}^{I}=s,a_{0}=a^{i}]\right],$$ (4)

where γ∈(0,1) is a discount factor, *I* is the identity function and i∈{A,E}. These SRs are initialised using transition matrices under the uniform policy and then are updated online using TD-learning.

The initialisation is according to allocentric and egocentric transition matrices that are created from sample trajectories. Allocentric samples come from following a uniformly random choice of allocentric actions in an empty environment. Egocentric samples come from following a uniformly random choice of egocentric actions in a random environment with barriers.

The TD learning rule has long been used to learn the standard state-state successor representation,

$$M_{s}^{i}[\phi |s]={𝔼}_{\pi }\left[\sum_{t=0}^{\infty }\gamma_{i}^{t}I[s_{t}^{I}=\phi |s_{0}^{I}=s]\right],$$ (5)

in an online manner [14]. This learning rule is, after seeing the state transition $\left(s,s^{\prime }\right)$, given by

$$M_{s}^{i}[\phi |s]\leftarrow M_{s}^{i}[\phi |s]+\alpha_{i}\left(1[s^{\prime }=\phi ]+\gamma_{I}M_{s}^{i}[\cdot |s^{\prime }]-M_{s}^{i}[\cdot |s]\right).$$ (6)

The state-action-state SR can be simply derived from the above by noting that it is the corresponding state-state SR conditioned on *a*_0_ = *a*, i.e.

$$M^{i}[\phi |s,a^{i}]={𝔼}_{\pi }\left[\sum_{t=0}^{\infty }\gamma_{i}^{t}I[s_{t}^{I}=\phi |s_{0}^{I}=s,a_{0}=a^{i}]\right]$$ (7)

$$=𝔼\left[{𝔼}_{\pi }\left[\sum_{t=0}^{\infty }\gamma_{i}^{t}I[s_{t}^{I}=\phi |s_{0}^{I}=s]\right]|a_{0}=a^{i}\right]$$ (8)

$$=𝔼\left[M_{s}^{i}[\phi |s]|a_{0}=a^{i}\right].$$ (9)

We therefore derive the update rule for the state-action-state SR by conditioning both sides of Eq 6, to give us, after observing a transition $\left(s,a,r,s^{\prime }\right)$,

$$M^{i}[\phi |s,a^{i}]\leftarrow M^{i}[\phi |s,a^{i}]+\alpha_{i}\left(1[s^{\prime }=\phi ]+\gamma_{i}M_{s}^{i}[\phi |s^{\prime }]-M^{i}[\phi |s,a^{i}]\right).$$ (10)

Here $\alpha_{i}$ is the SR learning rate and $1[s^{\prime }=\phi ]$ corresponds to a one-hot encoding of state $s^{\prime }$.

The model concurrently uses Eqs 6 and 10 to update cached state-state and state-action-state SRs.

Note that the allocentric transition graph is larger than the egocentric one, but with sparser connections. Meanwhile the egocentric transition graph has fewer nodes, because of aliasing, but with denser connections. Therefore the SRs have very different magnitudes. This motivated the use of ADAM as a means to adapt the learning rates appropriately for both bases. A comparison between an agent without ADAM and the full agent is shown in S3 Fig.

### 2.5 Value function

Concurrently, the agent learns a state-action value function using linear function approximation. The agent’s *Q*-values are

$$Q(s,a)=𝐰\cdot [M^{A}[\cdot |s^{A},R_{d}^{-1}(a)],M^{E}[\cdot |s^{E},a]],\;s=(s^{A},s^{E}),\;𝐰\in {\mathbb{R}}^{N^{A}+N^{E}}\;a\in {𝒜}^{E}.$$ (11)

Here $N^{A}=C_{A}$ and $N^{E}\leq C_{E}$ respectively denote the dimensions of the allocentric and egocentric state spaces, with *C*_*A*_ being the size of the gridworld and $C_{E}=2^{(H+1)(2H+1)-1}$, where *H* is the horizon.

The allocentric SR uses the allocentric action set ${𝒜}^{A}$ which are then transformed into the corresponding egocentric actions using the head-direction of the agent.

The weight vector **w** is learned using *Q*-learning with ADAM, such that after observing the $t^{\text{th}}$ transition $\left(s,a,r,s^{\prime }\right)$ the update is

$$𝐠=\left(r+\gamma \max_{a^{\prime }}Q(s^{\prime },a^{\prime })-Q(s,a)\right)\cdot [M^{A}[\cdot |s^{A},R_{d}^{-1}(a)],M^{E}[\cdot |s^{E},a]]$$ (12)

$$𝐦\leftarrow \beta_{1}𝐦+(1-\beta_{1})𝐠$$ (13)

$$v_{i}\leftarrow \beta_{2}v_{i}+(1-\beta_{2})g_{i}^{2}\;i\in \left\{1,\ldots ,N^{A}+N^{E}\right\}$$ (14)

$$\hat{𝐦}=\frac{𝐦}{1-\beta_{1}^{t}}$$ (15)

$$\hat{𝐯}=\frac{𝐯}{1-\beta_{2}^{t}}$$ (16)

$$w_{i}\leftarrow w_{i}+\eta \frac{{\hat{m}}_{i}}{\sqrt{{\hat{v}}_{i}}}\;i\in \left\{1,\ldots ,N^{A}+N^{E}\right\}$$ (17)

t←t+1. (18)

All model parameters used for the experiments are shown in S1 Table.

### 2.6 Plotting of egocentric SRs

**Fig 2:**
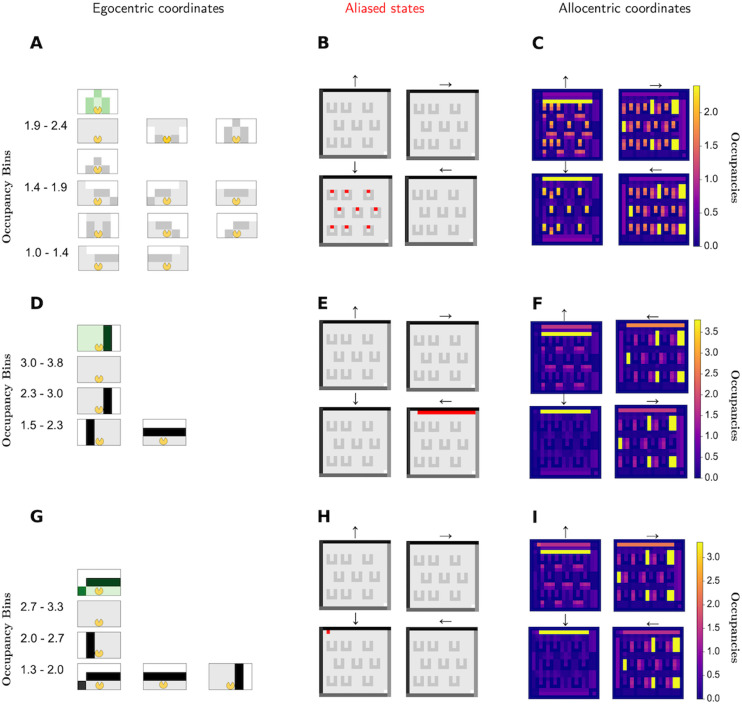
Egocentric state-state successor representations. **A-I** Three example egocentric state-state SRs plotted in both egocentric (A;D;G) and allocentric (C;F;I) reference frames. Note how the SRs look clearly different when visualised in egocentric coordinates but are similar when visualised in allocentric coordinates. The aliased (same egocentric state at different allocentric locations) locations of each egocentric state are shown in B;E;H. The visualisation in egocentric coordinates involves binning the SR vector into 5 equal occupancy bins and then displaying the egocentric states associated with the top 3 bins, in descending rows, so that the highest occupancy egocentric states are displayed in the top row, and so on. For further clarity, we also denote the starting state *s* in green above the rows. The occupancy bins are shown to the left of the corresponding states. The allocentric visualisation is similar to how we visualised standard allocentric SRs, with pixel intensity corresponding to the expected future occupancy of the state at that location. Since the egocentric state at a certain location is head-direction dependent, we now have four maps, one for each head direction, with the pixel intensity denoting similarly as the discounted expected future occupancy of that egocentric state. While the three SRs are difficult to distinguish when visualised in allocentric coordinates **(C, F, I)**, when visualised in egocentric coordinates they are clearly understandable as the expected future occupancies facing a barrier **(A)**, following a wall **(D)** and facing a corner **(G)**.

In Fig 2, three example egocentric SRs are plotted in both egocentric (left column) and allocentric (right column) coordinates. These come from just before the first world switch. Egocentric coordinates are associated with the (obscured) forward view of the agent. Thus, to show the egocentric SR is to show which particular forward views are prevalent following a given location and head direction, by averaging over future egocentric ’pictures’. When plotting in egocentric coordinates, we only display the states in the top 60% of occupancies. The numbers on the left hand side refer to the occupancy bin that the occupancies of the egocentric states to the right fall within. Fig 2B, 2E and 2H highlight in red the aliasing of those egocentric states across the first environment.

In Fig 2C, 2F and 2I, the same egocentric SRs are plotted in allocentric coordinates. Here, there are four heatmaps, each corresponding to a direction. The pixel colour at position (*x*,*y*) of heatmap *d* corresponds to the expected future occupancy of the egocentric state associated with position (*x*,*y*) and direction *d*. Due to aliasing of related states, the SR largely consists of blocks of colours where all the allocentric states with head direction correspond to the same egocentric state. Note the horizontal bars in Fig 2C. In the first world, all the barriers are oriented downwards with their exits at the top. Therefore in the SR in this environment, there is an association between the egocentric state at the opening to the barrier, and the egocentric state when facing the top wall, more than other walls. This then leads to the horizontal bars in the plot.

The egocentric states for which the SRs are shown correspond to barriers, walls and corners. Note how it might be hard to distinguish Fig 2C, 2F and 2I, but it is easy to distinguish Fig 2A, 2D and 2G. This shows how it difficult to disambiguate these distinct egocentric states by plotting their allocentric frequencies. However, once visualised egocentrically, disambiguation is more straightforward.

### 2.7 Multiple task comparisons

For the multiple task comparison in which we test ablations of the model (Fig 5), we run the agent in a collection of paradigms, each one which randomly samples worlds from different generative models. The generative models either generate barriers with fixed sizes or differing sizes and either fix the orientation of the different barriers or orient each barrier randomly (results shown in Fig 5A, 5B and 5C). We also employ a further generative model that generates fully-random non-compositional gridworlds (results shown in Fig 5D).

### 2.8 GLM analyses

#### 2.8.1 Barrier GLM.

For each task seed we fit a Poisson regression GLM to predict the mean steps in each world from the number of barriers (which we use the number of coloured pixels as a proxy). The equation being fitted is

$$\log 𝔼[\text{mean steps}]=\beta_{0}+\beta_{1}\cdot \text{barrier count}.$$ (19)

We then plot the mean $\beta_{1}$ and error bars denote standard deviation in S6 Fig. This is plotted for both full and non-egocentric (i.e. lesioned) agents.

#### 2.8.2 Occupancy GLM.

To determine the critical factors that distinguish the full versus the lesioned agent, we fit a logistic GLM on occupancy proportions from single episodes in each chunk of 25 episodes. We denote *f*_*walls*_ as proportion spent near walls, and *f*_*current*_ as proportion of time in current barrier locations, and *f*_*k*_ for proportion of time in locations where barriers were *k* worlds before. The equation being fitted is

$$\text{logit}P(\text{agent is lesioned})=\beta_{0}+\beta_{walls}f_{walls}+\beta_{current}f_{current}+\sum_{k=1}^{\text{num. worlds seen}}\beta_{k}f_{k}.$$ (20)

The significant (*p* < 0.05) coefficients from this analysis are shown in (S5 Fig).

### 2.9 Random world generation

- Barrier experiments. Each task consists of 5 random gridworlds, with rewards alternating between top-right and bottom-right corners. The barriers are generated by randomly sampling a top-left coordinate of the barrier, and then inserting a U-shaped block if a one-pixel buffer around it is clear. This procedure is repeated 20 times. In the main experiment, the first world barriers are constrained to be oriented in the same direction and then this constraint is lifted for the other worlds. For the other barrier experiments, it is the same procedure, but with differences in barrier size and alignment.
- Randomly generated non-structured world. Each world is generated by starting with an initially empty interior and then scattering single-pixel obstacles until a target density (0.2) is reached. Every candidate obstacle keeps the reward reachable and preserves connectivity; otherwise it is discarded. Connectivity preservation is checked by, after each addition of an obstacle, implementing a breadth-first search of all reachable states from a point at the opposite corner of reward, and comparing this set to the set of non-obstacle states. If either these two differ then we consider connectivity preservation to have been violated.

## 3 Results

**Fig 3:**
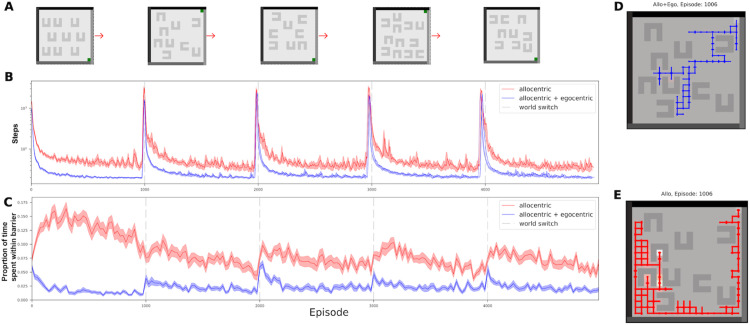
Lesion comparison - multi world learning. **(A)** A sample task, in each task we randomly sample all given worlds, with the first world having all the barriers oriented the same way, and no restriction in the other four. **(B)** Comparison of two models in the task, averaged over 30 different tasks. One model (blue) is the full allocentric+egocentric agent and the other (red) is a purely allocentric agent, without an egocentric component. The egocentric SR aids the learning of optimal strategies - although both step counts spike at environment switches, the full agent quickly relearns optimal strategies, while the lesioned agent is much slower. **(C)** Plots of the average time spent inside the barriers, for the full and egocentrically lesioned agents, calculated over 30 different random task generations. One can see that the full agent has learned to avoid the barriers, while the lesioned is slower to, and less good at it – demonstrating differences in generalisation. **(D, E)** Sample paths taken in the the second environment of a task, soon after an environment switch. **(D)** shows an example path taken by the full agent. It avoids the barriers and gets to the reward quickly. **(E)** shows an example path taken by the lesioned agent at the same point in training. It can be seen to get caught in the new barriers.

We built an agent which acquires both egocentric and allocentric state-action successor representations, and uses them as the basis for learning *Q*-values to navigate to a goal amidst scattered obstacles. The locations of the goal and the obstacles change every 1000 episodes. A sequence of 5 environments is shown in Fig 3A.

### 3.1 Egocentric SR facilitates generalisation

Our agent uses an egocentric successor representation (SR) to encode short-range, direction-dependent predictions of future egocentric state occupancy relative to nearby local structure (walls and barriers), alongside a conventional allocentric SR that captures a form of path distance to reward. The SR is a predictive representation which allows the agent to generalize lower value to headings whose predicted near-future visitation runs into barriers, and higher value to headings that safely skirt boundaries toward the goal. Because local egocentric structure (e.g., “a wall directly ahead at this angle”) recurs across different layouts, these egocentric predictions transfer when the world changes, even when the global structure does not.

We evaluate this egocentric agent in a multi-world task generated from a common task distribution. Each task consists of repeated environment switches: barrier layouts and reward locations change, preserving only local regularities that the egocentric SR can recognise. Performance is summarised by (i) mean steps to reach reward across tasks at different episodes, (ii) mean time spent inside barriers (a direct measure of collision/trapping), and (iii) representative trajectories taken shortly after a switch.

The results of a comparison between the agent and a “lesioned” agent without an egocentric component are shown in Fig 3. It can be seen that the agent rapidly learns to find the goal with efficient paths. Furthermore, having had experience with the barriers, it spends little time being trapped by them. The agent has no capacity to meta-learn, and so had to re-adapt to the change each time in happens (hence the spikes in performance every 1000 episodes in Fig 3B). However, the capacity to avoid getting trapped in barriers generalized well after environmental changes because of the local consistency of the different worlds in egocentric terms, even though the global structure had changed. This meant that even straight after a change, the barriers could be duly avoided, as shown by the sample path in blue (Fig 3D).

The “lesioned” agent, in contrast, takes longer paths and the obstacles are more of a consistent hazard. Furthermore, the lesioned agent avoided empty areas of the world where barriers were previously situated (S5 Fig), leading to inefficient strategies. We optimized our lesioned baseline maximally by optimizing the shared hyperparameters between the lesioned and unlesioned agents to maximize lesioned agent performance and then only optimized egocentric hyperparameters on top for the unlesioned agent performance. We also compare (S4 Fig) an agent that just uses the egocentric state space for *Q*-learning rather than the egocentric SR, this agent hardly does better than the fully lesioned agent, highlighting the importance of the predictive representation. This shows some of the numerous benefits of having an egocentric representation. For completeness, we also plotted the performance of an egocentric-only agent (S2 Fig).

### 3.2 The agent learns compositional value functions that facilitate efficient continual learning

The full agent represents state value compositionally as the sum of two learned terms that factor different structure in the task pertaining to different reference frames. Formally, with state (s=(x,y,d)) (position and head-direction) and ($V(s)=\max_{a}Q(s,a)$), the learned value is linear in its features, so the allocentric and egocentric contributions can be separated and plotted independently:

$$V(x,y,d)=V^{a}(x,y)+V^{e}(x,y,d)$$ (21)

$${\bar{V}}^{e}(x,y)={𝔼}_{d}V^{e}(x,y,d).$$ (22)

**Fig 4:**
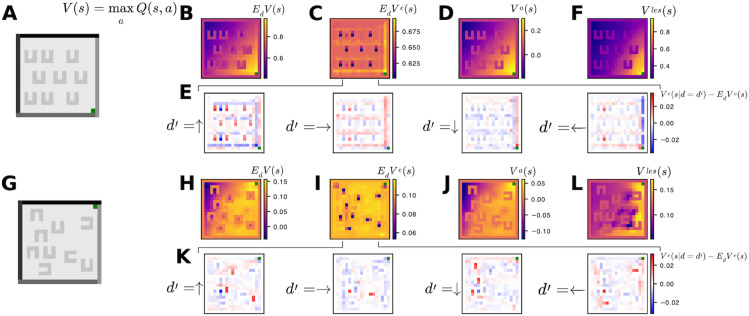
Compositional value functions and transfer. **(A)** Example first world, with reward (green box) walls and barriers. **(B)** Learned state value function ($V(s)=\max_{a}Q(s,a)$) learned by the agent at the end of training in the first world, averaged uniformly over head-directions. **(C)** Egocentric component ${\bar{V}}^{e}(x,y)={𝔼}_{d}V(x,y,d)$: local perturbations around barriers and walls. **(D)** Allocentric component *V^a^*(*x*,*y*): a smooth gradient toward reward that largely ignores barriers. **(E)** Direction-dependent egocentric residuals ($V^{e}(x,y,d)\;-\;{\bar{V}}^{e}(x,y)$): preferences for headings that follow reward-leading walls and avoid barrier concavities. **(F)** Lesioned agent value function: it learns a similar value function but in a non-compositional manner. **(G)** New environment after switch. **(H-K)** Full agent value components shortly after the switch: positive value near the new reward and appropriately negative value around new barriers, with minimal artifacts from the old layout. **(L)** Lesioned agent after the switch: it has not learned the negative value of some of the new barrier locations and there are clear deleterious artifacts from the previous world. Walls and barriers are inaccessible; their pixels are rendered with the mean colour of accessible states.

We plot these different contributions in Fig 4. Fig 4B, 4C, 4D, 4E and 4F show the different contributions to the value function that are learned at the end of learning in the first environment, shown in Fig 4A. The allocentric map (*V^a^*(*x*,*y*)) forms a smooth gradient that increases toward the reward location and is largely insensitive to barriers. This produces the right-down global bias (to move up the gradient toward the reward) but on its own would encourage the agent to get trapped behind obstacles. The egocentric term (*V^e^*(*x*,*y*,*d*)) fixes this by learning local, subtractive penalties tied to head-direction and nearby observations: it depresses value when the current heading would drive the agent into a wall or barrier, and also learns egocentric wall-following strategies. The lesioned control that lacks the egocentric term still learns the reward gradient (via (*V^a^*)) and thus solves the training environment at steady state, but the it does not learn reusable strategies.

When the walls and barriers are rearranged and the reward relocates, the contrast is starkly apparent after only a small amount of new experience. The full agent rapidly suppresses value around the new barrier locations and assigns high value near the new reward location. This happens because the egocentric features encode local patterns that recur across worlds; the previously learned egocentric penalties immediately apply to the new layout and only need light retuning. By comparison, the lesioned agent exhibits deleterious artifacts at old barrier sites and missed penalties at new ones: its allocentric gradient can move to the new reward, but without egocentric corrections it both overgeneralises obsolete structure and undergeneralises to new barriers (S5 Fig). The factorisation in the full agent’s value function is what enables efficient continual learning. The global map adapts to a moved reward, while the local egocentric strategies transfer across worlds that share similar local structure.

### 3.3 Contribution of egocentric component varies with the local consistency in egocentric terms of the different worlds

**Fig 5:**
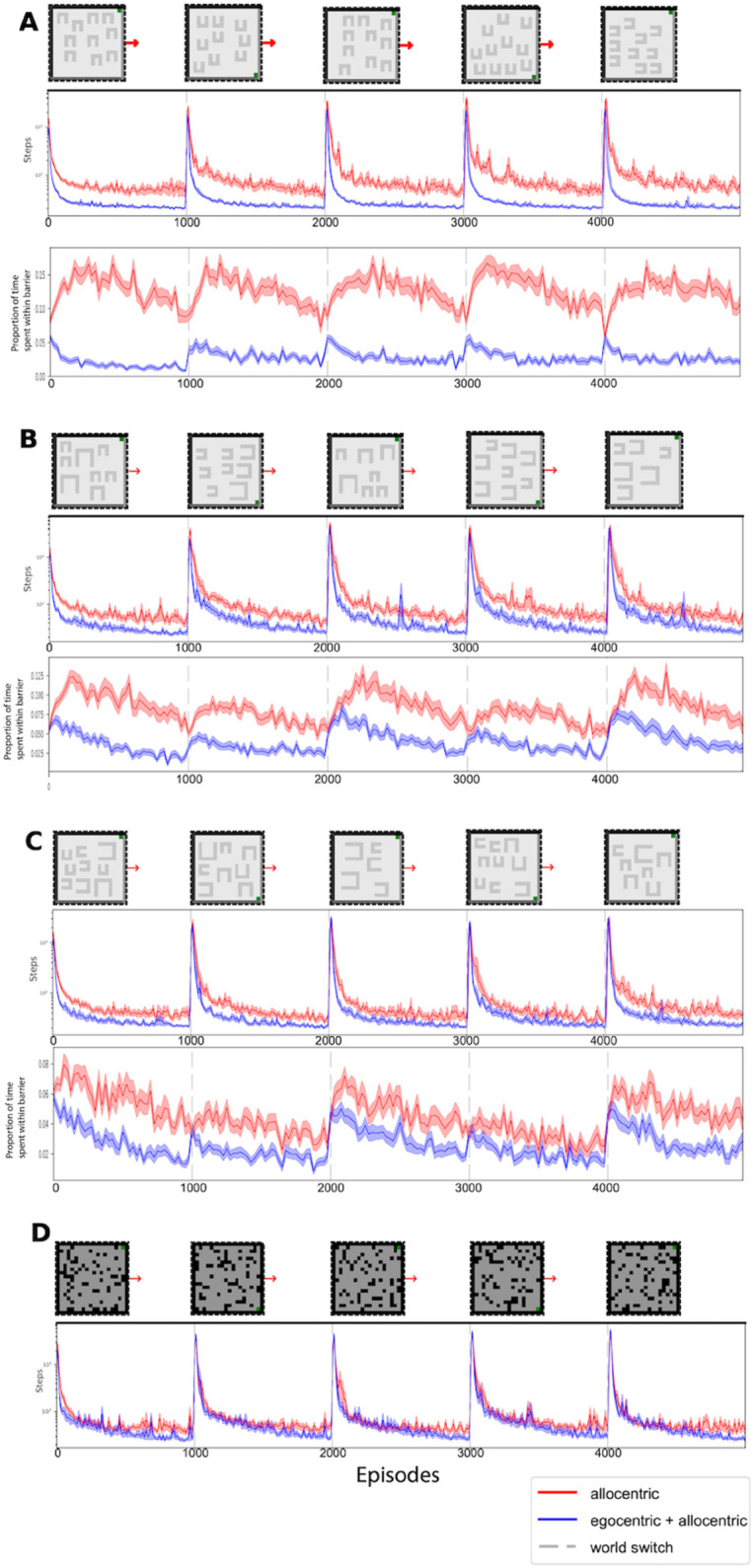
Comparison of the benefits of the egocentric component in other task paradigms. **(A-D)** Comparisons between full (allocentric + egocentric) and lesioned (allocentric-only) agents across paradigms where tasks are being sampled from different generative models. Each plot consists of (on top) an indicative sample task, with example gridworlds and arrows in between to signal the world switches,(middle + bottom) the lesion steps (middle) and hole times (bottom) comparison across runs with different tasks drawn from a specified generative model. **(A)** Comparison between full and lesioned agent where environments consist of randomly generated barriers, of the same size, which are all aligned to obstruct reward access. **(B)** Comparison between full and lesioned agent where environments consist of randomly generated different-sized barriers which are all aligned to obstruct reward access. **(C)** Comparison between full and lesioned agent where environments consist of randomly generated different-sized barriers which are all randomly orientated. **(D)** Comparison between full and lesioned agent where environments consist of randomly generated worlds with no shared egocentric structure across environments. The black pixels represent barriers. The hole times plot is excluded here as there is no concept of holes in these fully random gridworlds.

In order to investigate the variability in the utility of the egocentric representation across a range of task-types, we re-ran the lesion comparison in tasks with differing levels of local consistency. We compared full and allocentric-only agents in different tasks where the worlds were sampled from generative models that either kept the barriers aligned against reward (Fig 5A and 5B), or where orientations were randomly chosen per barrier (Fig 5C). We also did this for worlds where the barriers could change their size (Fig 5B and 5C). We then ran the agent in fully randomly generated worlds where the only restriction was that space had to be fully-connected (Fig 5D).

We found a gradient in the differences in performance between the full and lesioned agents that depends on the consistency of local egocentric structure as well as the usefulness of knowledge of such structure. However, inclusion of the egocentric SR proved to be beneficial in the tasks investigated. This can also be seen by plotting the ratios of the number of steps taken by the unlesioned and lesioned agents, the histograms of which are shown in S7 Fig. One could also imagine structures where it would not necessarily be beneficial, such as if suddenly rewards were inside the barriers.

We also re-ran the analysis in the main paper for different horizon values H=3,4,5 (optimising egocentric parameters for each horizon separately). We found that the advantage of the egocentric agent decreased with larger horizon values, presumably due to the reduced amount of aliasing and the greatly increased size of the egocentric state space. The plot of mean-steps-taken against horizon value is shown in S1 Fig. The aliasing associated with a small horizon has a beneficial effect for generalization. Our expectation that this would be maintained for larger horizons by the use of the successor representation, which could link non-aliased but similar states, was not bourne out. However, we constructed the egocentric SRs based on naive pixel input; representations based on more abstract (but still egocentric) representations of the input might cope with a longer horizon.

## 4 Discussion

Our results highlight clear benefits of egocentric associative representations in complex spatial processing. We developed a model which combines a standard allocentric SR for global positioning with an egocentric SR which allows it to navigate efficiently and flexibly around multiple complex environments by exploiting their shared local structure. We demonstrated the benefit of such an egocentric SR by showing what happens when it is lesioned, and, thanks to the simple linear nature of the model, unpicked the contributions of the different components to the value function.

**Allocentric and egocentric representations:** Our understanding of the cognitive mechanisms of spatial processing has been dominated by approaches that are focused on allocentric representations. This is prompted by their evident neural signatures in the rodent hippocampal formation including place cells [4] and grid cells [31]. Lesion experiments further corroborate the hippocampal formation’s role in navigation [2]. Cognitive maps offer a unifying framework for such analyses, with both place cells and grid cells being seen as being key to understanding the neural basis of such a map [4,31,32].

Modelling approaches to understanding the role of the hippocampus in spatial processing also concentrate on allocentric representations. For instance, it has been suggested that the hippocampus offers a predictive map of the future [33], viewing place cells as the neural implementation of an allocentric SR and grid cells encoding a low-dimensional basis set from which to build such a representation. There have also been suggestions that the hippocampal formation performs latent allocentric structure learning on egocentric inputs [20,21] with grid cell and place cell properties being explained by various functions including path-integration [32,34,35] and probabilistic message-passing [36]. Some of these approaches view allocentric representations such as place cells as latent states of a generative model that tries to reconstruct egocentric sensory information [37–39].

By contrast, work on egocentric representations either focuses on translating from egocentric to allocentric representations, possibly a task for retrosplenial cortex [22,40], or, in navigational terms (e.g., [1]) tends to regard them as being used for simple taxon-based stimulus-response learning rather than the sort of planning and associative structure learning that is equated with allocentric representations. Unlike these proposals, our approach, although using RL and dealing with navigation rather than explicit prediction, suggests one should see them in similar terms to the cognitive map formulations described above, and suggests that when egocentric information is informative, we might expect the hippocampal latent code to capture it, along with allocentric information.

**Deictic representations:** Egocentric representations were used for planning in [27]. Drawing on work on visual routines [41], this made the distinction between capital-P “Planning”, where a smart Planning phase constructs a Plan which is carried out mechanically, and lowercase-p “planning” which is closer to recipes, which they claim is a better description of naturalistic human behaviour. [27] suggested deictic representations, indexical-functional entities such as “the-block-that-I-am-pushing”, as a means of overcoming the combinatorial explosion associated with “Planning” using propositions. Deixis has been extensively explored in linguistics [42], stemming from the introduction of terminologies such as indexicals, which are linguistic expressions whose reference can shift between contexts. The sort of policies learned by the agent in this study include “plans” based around deictic representations such as “the-barrier-that-I-am-facing”, rather than a plan based around an allocentric representation of the form “barrier1” (as distinct from “barrier2”, which might be physically identical, but placed somewhere else in the environment).

**Compositionality:** Our investigation shows how exploiting compositional representations can be of benefit in dynamic tasks, for instance facilitating continual learning. Our world model is factorized into simple egocentric and allocentric maps, allowing it to represent separately different dimensions of the environment and thereby generalize better to new environments. Indeed, we showed how performing spatial processing in an egocentric reference frame enables a form of passive generalisation across environments which share local structure. This led to fast learning after environmental change.

More generally, animals could learn to navigate complex environments by composing simple maps representing different reference frames. These different reference frames need not be egocentric and allocentric, but could rather reflect (possibly learned) priors over other important dimensions that collectively factorise a world model. For instance, evidence about object-based attention [43] suggests that objects might provide another useful reference frame that would be closely related to our egocentric suggestion.

Related modeling work on the hippocampus also makes use of compositional representations to facilitate generalisation. [44] employ a meta-learning approach to train a large neural network from pre-determined optimal policies, and thereby show how using state spaces which are composed from reward and object-vectors can help one infer optimal policies in new environments better than using pure spatial representations. Though similar in flavour, there are various differences with their approach, including our application of RL with a simple linear representation to show how a factored representation allows quicker learning and generalization in real-time.

Factorised approaches have also been successful in SLAM. The main original SLAM approaches were based on algorithms such as the Extended Kalman Filter, where a robot might navigate by maintaining and updating a mean vector (best estimate) and a covariance matrix (expected error) of the locations of itself and *N* landmarks. However, the computational complexity of this, mostly stemming from the quadratic nature of the covariance matrix, have more subsequently led to approaches that exploit local connectivity [45,46] or decompose the map into sub-maps [47] rather than learning full global structure. These updated approaches to navigation are conceptually similar to the approach we have advocated, by emphasising the importance of local coordination and representations that are factorised into simpler sub-maps – i.e., parallel learning mechanisms operating on local and global state representations.

**Hierarchical abstract machines (HAMs):** A separate motivation for our work was hierarchical abstract machines (HAMs; [29]), which were an influential early suggestion for hierarchical RL. In HAMs, modest-sized state machines act as partial, closed-loop polices. One motivation for HAMs was that one could compose an overall policy (getting to a goal efficiently) out of smaller parts (avoiding getting trapped in barriers). HAMs do not have to involve exclusively egocentric information – nevertheless, as we have seen, this can have great benefits in terms of generalisation. In sum, we can see our egocentric components adding a form of compositionality to the policy.

**Egocentric representations in the brain:** Our work was also influenced by the possibility that the brain might generate the same sort of relational structure for egocentric representations that it apparently does for allocentric ones [20,33,48,49]. There are numerous areas in the brain that might be implicated in this function. One candidate is the lateral entorhinal cortex (LEC), a key input to the hippocampus [50]. In the LEC there is some evidence for coding of external objects in an egocentric reference frame [51] and reward related egocentric encoding [52], with distinct populations of neurons representing reward approach, consumption and departure. Other possible regions include the parietal cortex or its targets implicated in transformations between egocentric and allocentric coordinates [53–55], as well as the PFC, where there is evidence for the coding of task structure in an egocentric reference frame [56,57]. It has also been found that the activities of a modest fraction of neurons in the hippocampus of fruit bats have some egocentric components [58], along with egocentric coding in rodents and primates for aspects of the environment such as landmarks or obstacles [59–61]. One should note the difficulty of characterising egocentric associative representations by looking at allocentric “rate” maps and indeed take into account the fact that the coordinate systems in which the egocentric SRs are transparent might exhibit extra complexities such as direction dependence. This is demonstrated in our analysis of egocentric SRs.

**Limitations and extensions:** In order to focus on the nature of the spatial representation, we considered a very simple problem and domain, with an environment that was deliberately designed to include shared local structure. Navigation in richer environments with more and different obstacles could benefit from non-linear functions of allocentric coordinates and egocentric input, as would arise from multi-layer neural networks. Other approaches have used multi-layer neural networks to train deep RL agents on egocentric pixel input to play FPS games [62]. These deep learning approaches have allowed agents to succeed in naturalistic tasks but the learned feature representations were not as amenable to investigation. Here we have made a case for representations that reflect the temporal structure underlying the raw egocentric inputs.

Extending the *R*_*d*_ function that aligns the reference frames to handle different relationships could be complex, for instance, with object-based reference frames requiring the alignment of rather different temporal scales of action. Our simple linear model demonstrates the development of simple egocentric strategies in navigational tasks, but is constrained by the necessity of close aliasing in the pixel space. A non-linear version of the model that could handle more naturalistic environments could be used to generate novel behavioural predictions regarding navigation strategies chosen by humans and animals using existing behavioural data. For instance, [63,64] show that humans and rodents are not always optimal in their path choices when navigating complex environments. [63] shows that human and rodent trajectories are better described by an allocentric-SR based agent than an optimal model-based agent – but it might be that including influences from an egocentric-SR based agent would fit the data better, with patterns of non-optimality being predicted by the egocentric structure of the respective tasks.

**Conclusion:** In sum, we have offered a perspective on egocentric contributions to spatial behaviour. An egocentric successor representation that reflects different information about spatial structure from the standard allocentric SR, can afford substantial advantages in suitable domains. Our results show how cognitive maps might represent different relational structures and how they could be combined to realize flexible cognition.

## Supporting information

S1 FigMean steps as a function of horizon.We re-ran our analysis with larger horizon values H=3,4,5, re-optimising the egocentric parameters for each horizon. As can be seen, the additional advantage that the egocentric component provides over the purely allocentric agent decreases as the horizon increases, presumably due to decreased aliasing across the environments and slower learning from the very much greater number of egocentric states.(PDF)

S2 Fig Full lesion comparison. Performance of the allocentric+egocentric, allocentric-only, and egocentric-only agents in the standard task paradigm. The egocentric-only agent performs poorly since its representation is local and does not contain sufficient information to find the reward.
(PDF)

S3 Fig Non-adaptive learning rate comparison. Performance of the full agent compared to an agent with no adaptive learning rate in the standard task paradigm. The non-adaptive agent performs poorly since it is unable to adapt its learning rate to the magnitudes of the two different bases.
(PDF)

S4 Fig Egocentric Q-learning comparison. Performance of the full agent compared with the lesioned agent and an agent that uses an allocentric SR but just does *Q*-learning on egocentric states instead of their associated successor representations. It can be seen that the “Egocentric *Q*-learner” does slightly better than the fully lesioned agent, but performs much worse than the agent equipped with an egocentric SR, highlighting the usefulness of the SR.
(PDF)

S5 Fig Chunked episode occupancy GLM. Timeseries of GLM coefficients. GLMs predicting agent type are fitted on post-first-switch occupancy proportions of trajectories, with separate regression coefficients for each chunk of 25 episodes. Different regressors are proportions of time spent near walls, within current barriers, and at previous barrier locations. One can see that the lesioned agent (in comparison the unlesioned agent) is associated with less time near walls, more time in current barriers, and there is a small effect of slightly less time in previous locations of barriers.
(PDF)

S6 Fig Barrier count GLM. Comparison of log-link coefficients of Poisson GLMs fitted to predict mean number of steps in a world using the number of barriers in that world, for both lesioned and unlesioned agents. Error bars show standard deviation of coefficients over 30 task seeds. We see that the mean effect of barrier count is slightly higher for the lesioned agent than the unlesioned agent but neither is statistically significant.
(PDF)

S7 Fig Ratios of steps taken. Histograms of the ratios of steps taken of unlesioned to lesioned agents per episode in the different random world types.
(PDF)

S1 Table Model hyperparameters. Model hyperparameters.
(PDF)
