# Supplementary figures and images for: Composing egocentric and allocentric maps for flexible navigation
Source: PLoS Comput Biol. 2026 Jan 23;22(1):e1013905. doi: 10.1371/journal.pcbi.1013905 (PMC12867328; doi:10.1371/journal.pcbi.1013905)

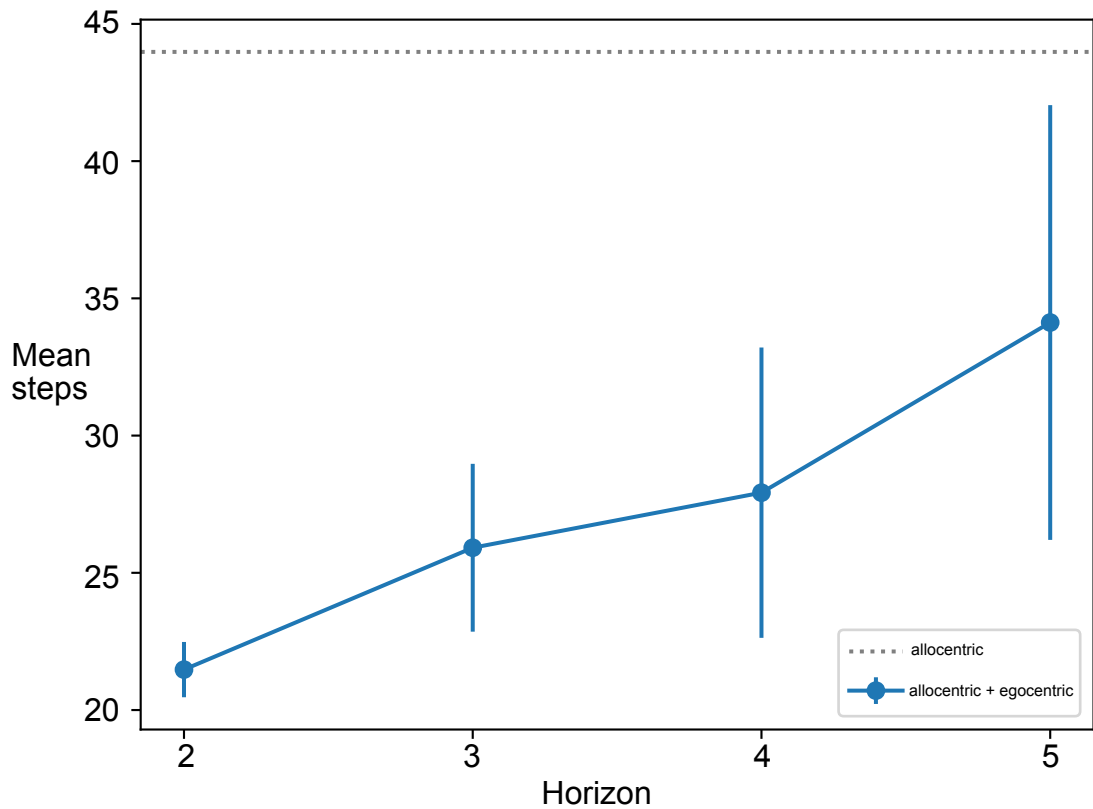

Supplement: S1 Fig — We re-ran our analysis with larger horizon values H=3,4,5, re-optimising the egocentric parameters for each horizon. As can be seen, the additional advantage that the egocentric component provides over the purely allocentric agent decreases as the horizon increases, presumably due to decreased aliasing across the environments and slower learning from the very much greater number of egocentric states. (PDF) [file pcbi.1013905.s001.pdf]

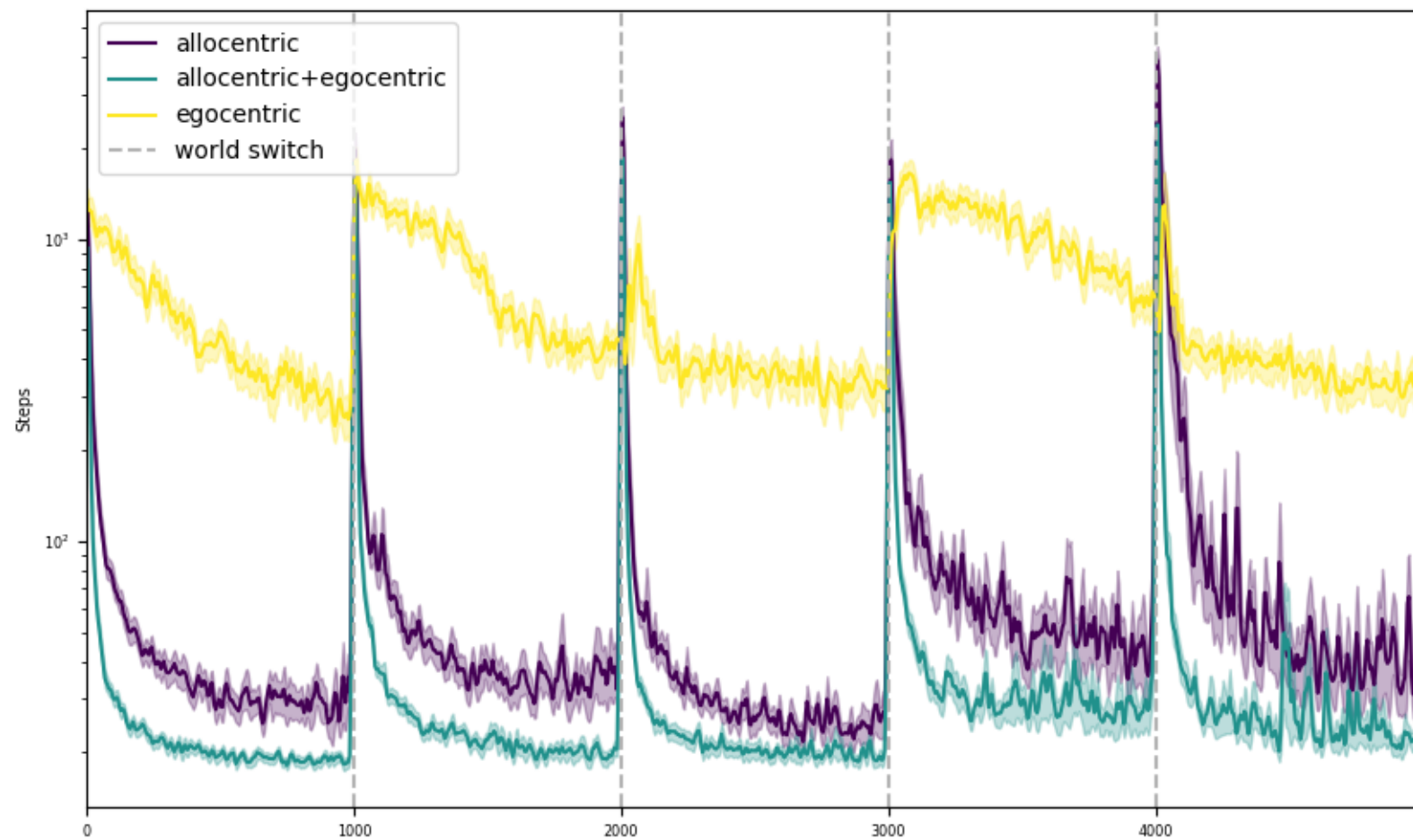

Supplement: S2 Fig — Performance of the allocentric+egocentric, allocentric-only, and egocentric-only agents in the standard task paradigm. The egocentric-only agent performs poorly since its representation is local and does not contain sufficient information to find the reward. (PDF) [file pcbi.1013905.s002.pdf]

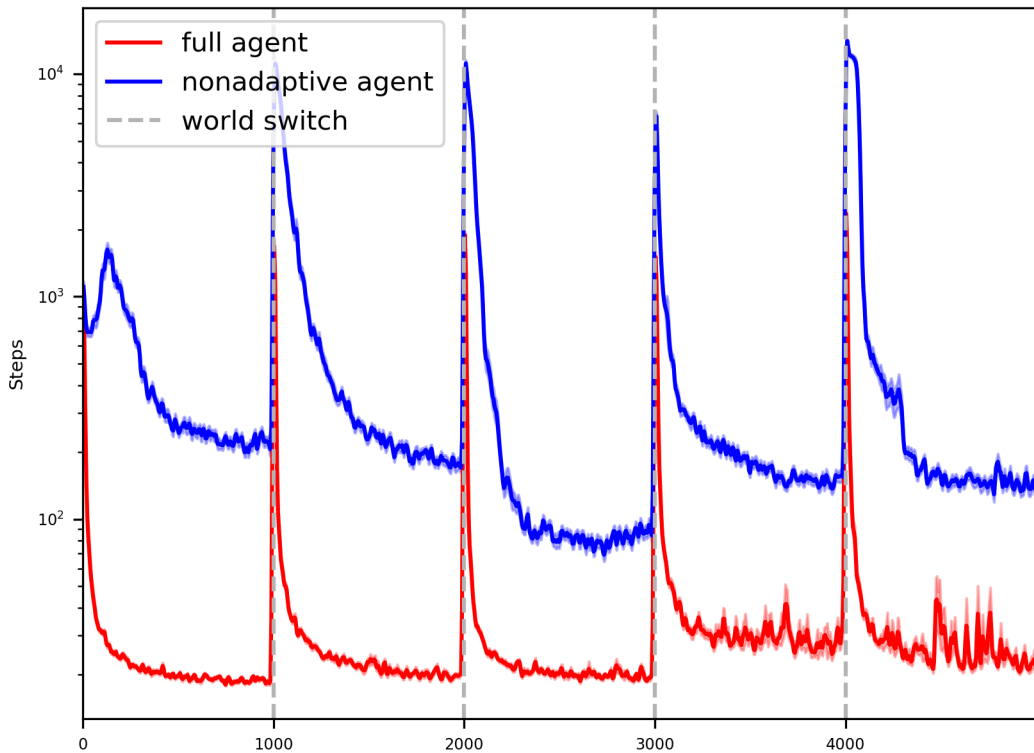

Supplement: S3 Fig — Performance of the full agent compared to an agent with no adaptive learning rate in the standard task paradigm. The non-adaptive agent performs poorly since it is unable to adapt its learning rate to the magnitudes of the two different bases. (PDF) [file pcbi.1013905.s003.pdf]

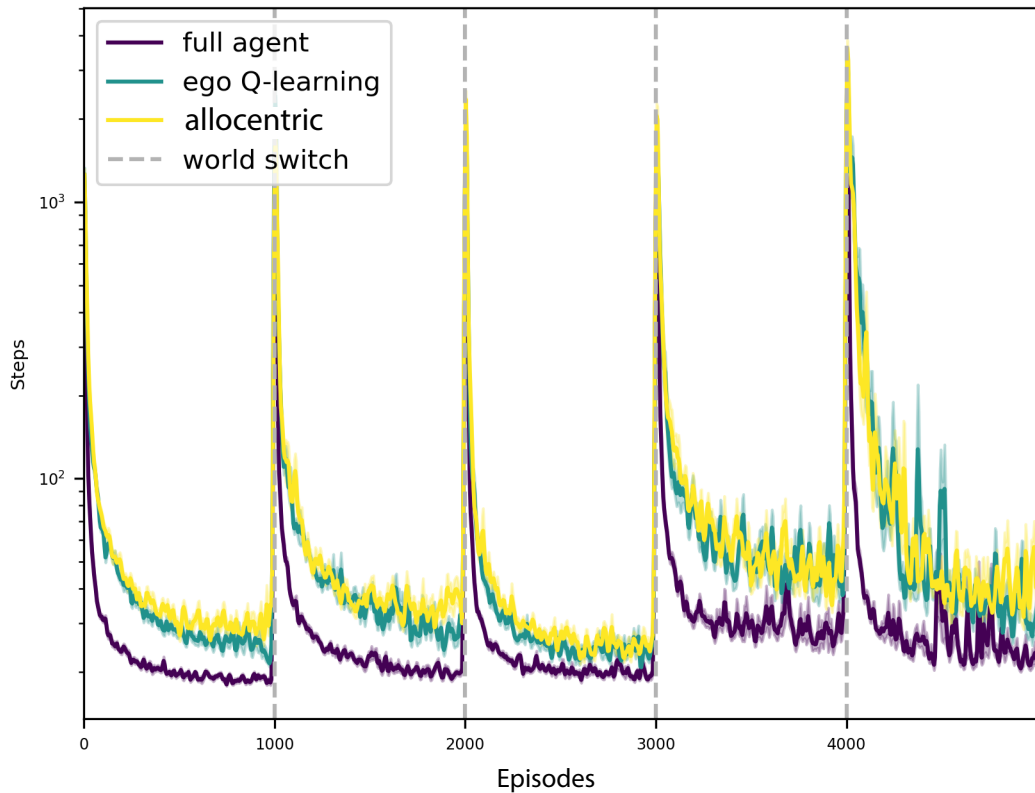

Supplement: S4 Fig — Performance of the full agent compared with the lesioned agent and an agent that uses an allocentric SR but just does Q-learning on egocentric states instead of their associated successor representations. It can be seen that the “Egocentric Q-learner” does slightly better than the fully lesioned agent, but performs much worse than the agent equipped with an egocentric SR, highlighting the usefulness of the SR. (PDF) [file pcbi.1013905.s004.pdf]

Episode-level GLM coefficients (significant only)  
 $p < 0.05$

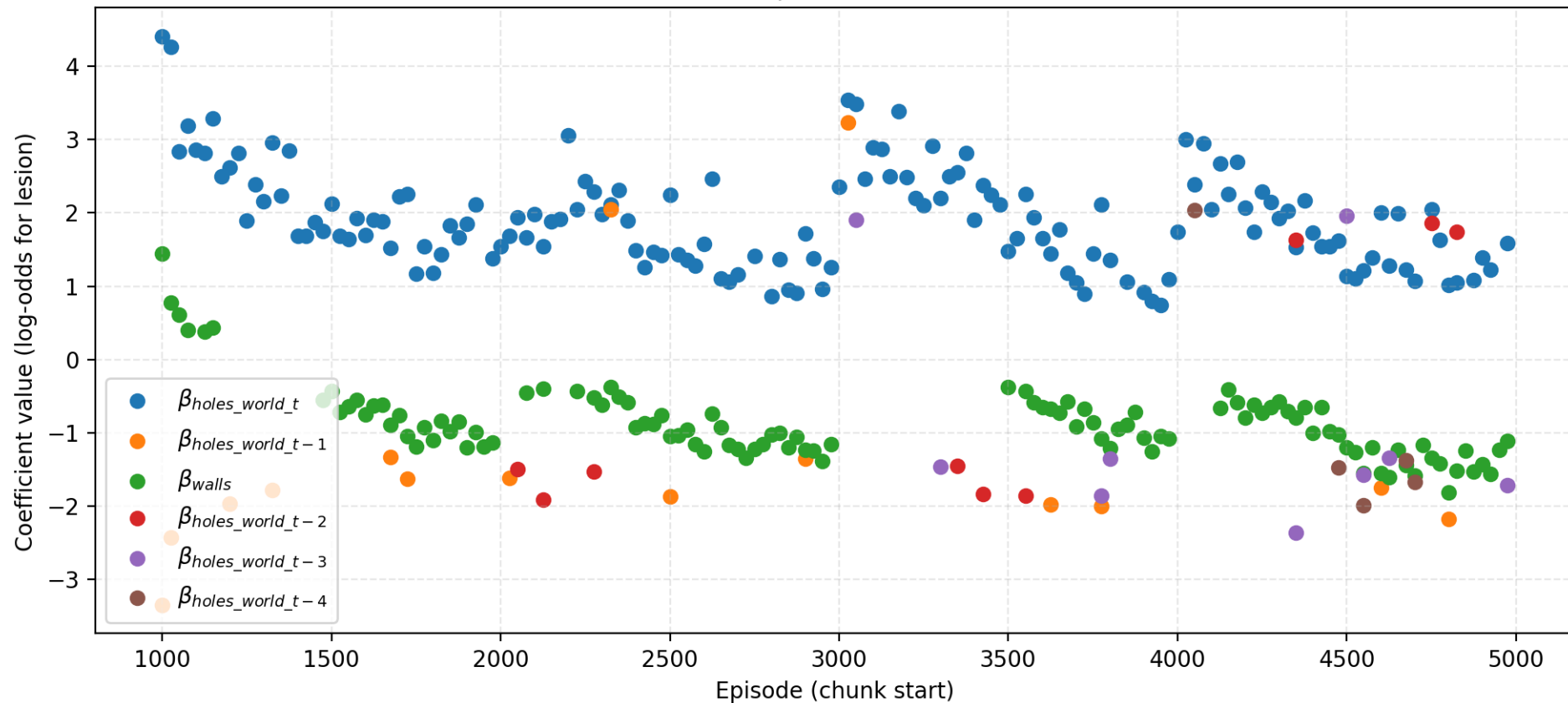

Supplement: S5 Fig — Timeseries of GLM coefficients. GLMs predicting agent type are fitted on post-first-switch occupancy proportions of trajectories, with separate regression coefficients for each chunk of 25 episodes. Different regressors are proportions of time spent near walls, within current barriers, and at previous barrier locations. One can see that the lesioned agent (in comparison the unlesioned agent) is associated with less time near walls, more time in current barriers, and there is a small effect of slightly less time in previous locations of barriers. (PDF) [file pcbi.1013905.s005.pdf]

Barrier GLM coefficients by agent condition

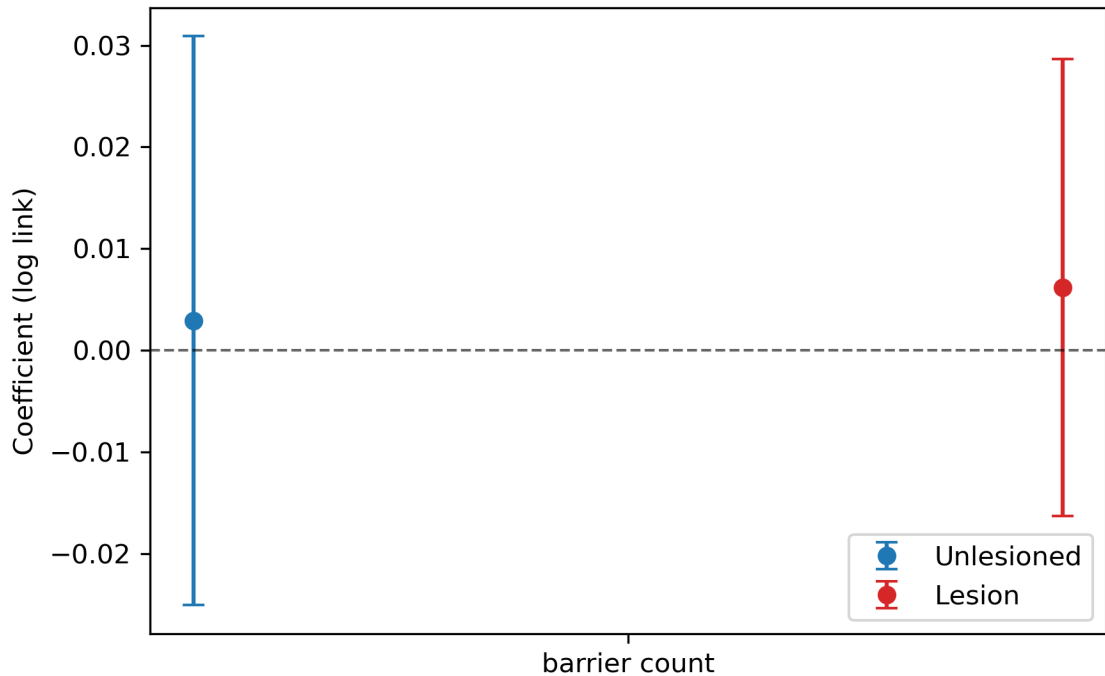

Supplement: S6 Fig — Comparison of log-link coefficients of Poisson GLMs fitted to predict mean number of steps in a world using the number of barriers in that world, for both lesioned and unlesioned agents. Error bars show standard deviation of coefficients over 30 task seeds. We see that the mean effect of barrier count is slightly higher for the lesioned agent than the unlesioned agent but neither is statistically significant. (PDF) [file pcbi.1013905.s006.pdf]

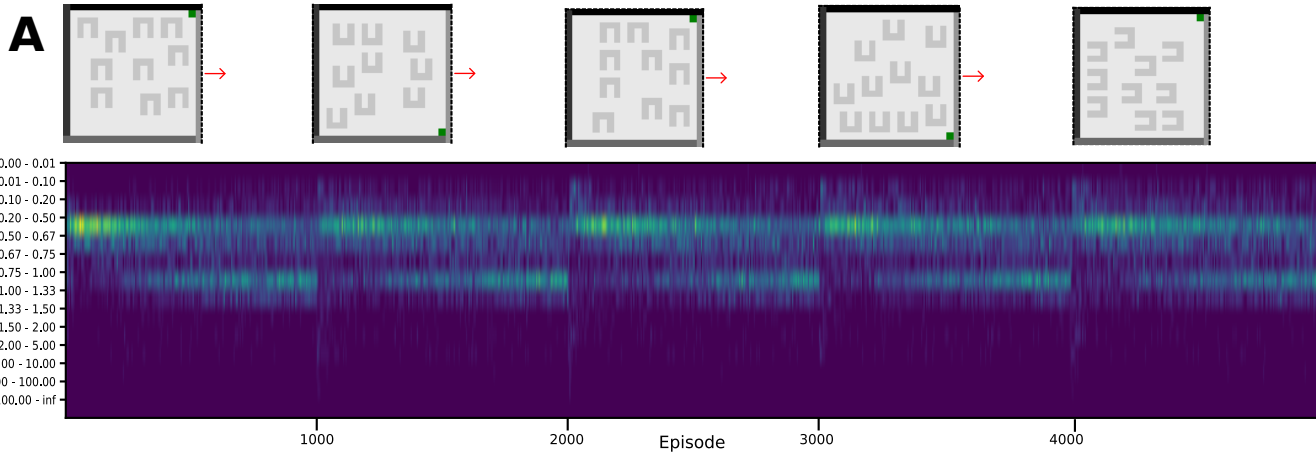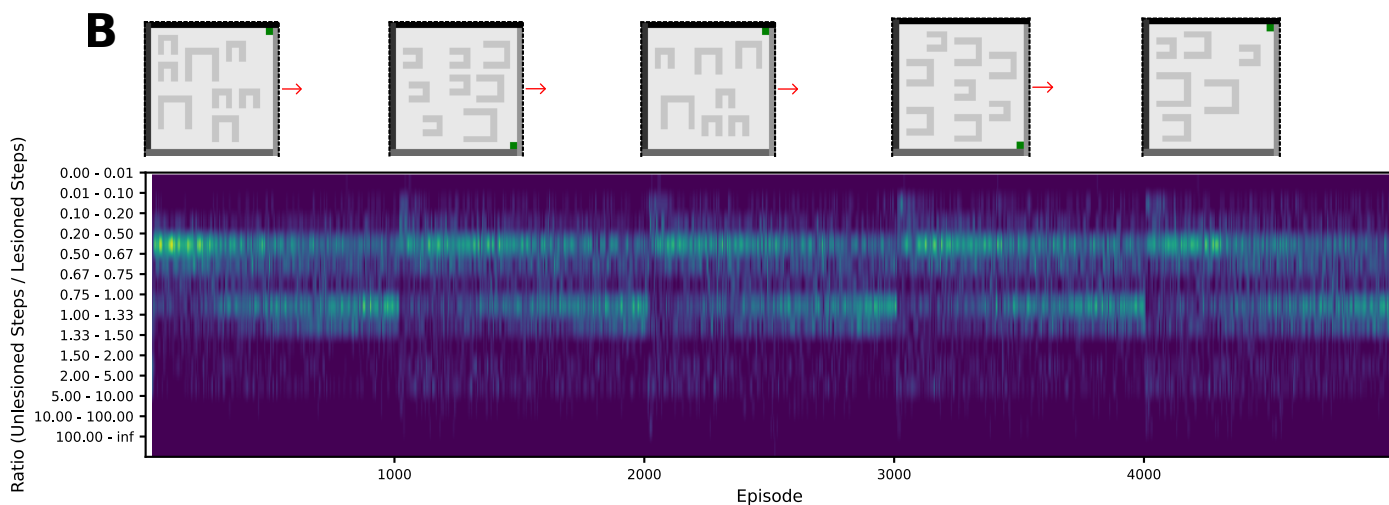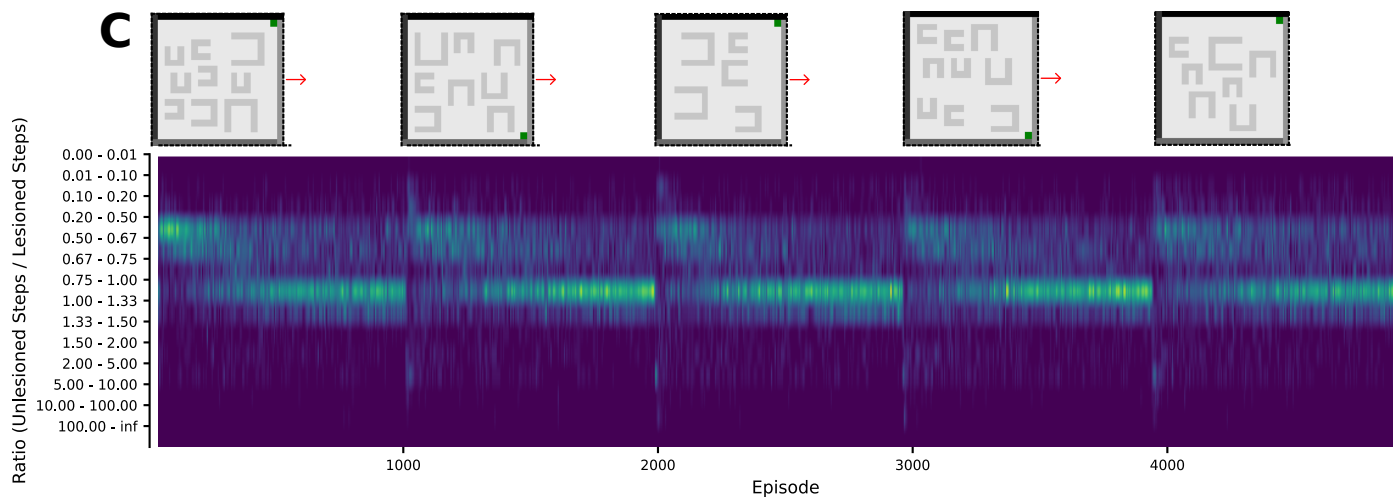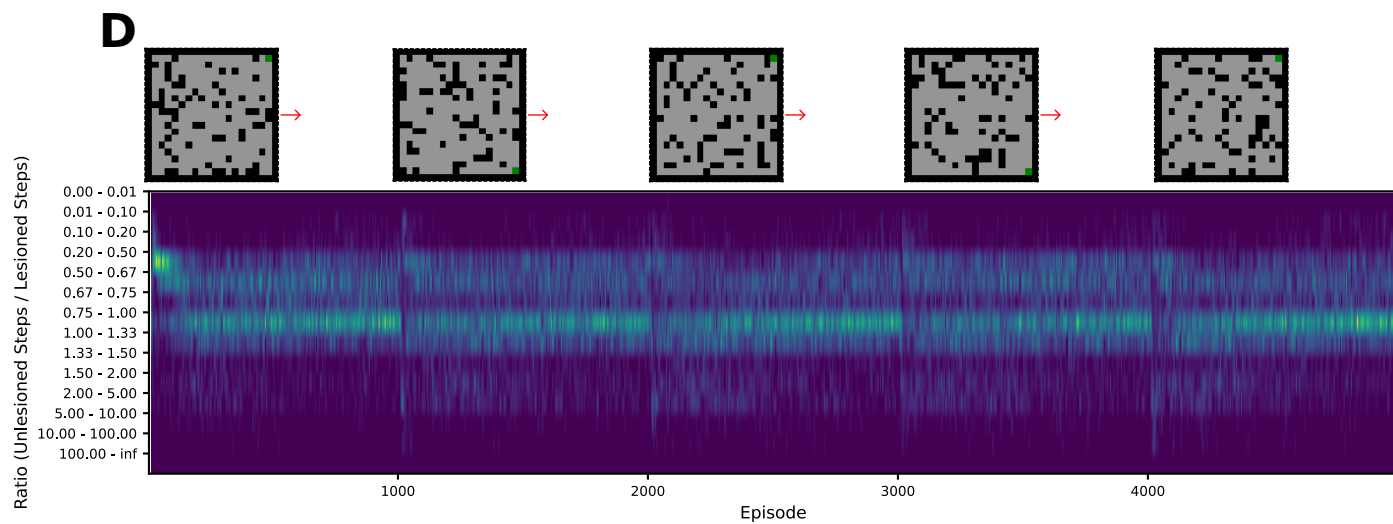

Supplement: S7 Fig — Histograms of the ratios of steps taken of unlesioned to lesioned agents per episode in the different random world types. (PDF) [file pcbi.1013905.s007.pdf]
