# Supplementary material for: Composing egocentric and allocentric maps for flexible navigation
Source: PLoS Comput Biol. 2026 Jan 23;22(1):e1013905. doi: 10.1371/journal.pcbi.1013905 (PMC12867328; doi:10.1371/journal.pcbi.1013905)
Supplement: S1 Table — Model hyperparameters. (PDF) [file pcbi.1013905.s008.pdf]

γ	0.98
γ_E	0.98
γ_A	0.94
α_E	0.035
α_A	0.0078125
τ	0.01
ϵ	0.01
β_1	0.9
β_2	0.999
